# Local anesthesia underutilized for inguinal hernia repair in northern Ghana

**DOI:** 10.1371/journal.pone.0206465

**Published:** 2018-11-21

**Authors:** Stephen Tabiri, Katie W. Russell, Frank E. Gyamfi, Ali Jalali, Raymond R. Price, Micah G. Katz

**Affiliations:** 1 School of Medicine and Health Sciences, University for Development Studies and Tamale Teaching Hospital, Tamale, Northern Region, Ghana; 2 University of Utah Department of Surgery, Center for Global Surgery, Salt Lake City, Utah, United States of America; 3 Holy Family Hospital, Berekum, Brong-Ahafo Region, Ghana; 4 University of Utah Department of Economics, Health Economics Core, Population Health Research Foundation, Salt Lake City, Utah, United States of America; University of Alabama at Birmingham, UNITED STATES

## Abstract

**Introduction:**

Inguinal hernia repair is a common procedure and a priority for public health efforts in Ghana. It is essential that inguinal hernia repair be performed in a safe, efficient manner to justify its widespread use. Local anesthesia has many favorable properties and has been shown to be superior, compared to regional or general anesthesia, in terms of pain control, safety profile, cost-effectiveness, resources required, and time to discharge. Local anesthesia is recommended for open repair of reducible hernias, provided clinician experience, by multiple international guidelines. Regional anesthesia is associated with myocardial infarction and other complications, and its use is discouraged by multiple guidelines, especially in older patients. This study aims to assess the current state of anesthesia for inguinal hernia repair in the northern and transitional zone of Ghana. In addition we will assess the perceptions of different types of anesthesia along with understanding of evidence-based guidelines among clinicians participating in inguinal hernia repair.

**Methods:**

We performed a retrospective review of all inguinal hernia repairs for male patients, 18 and older, in over 90% of hospitals in northern Ghana. All 41 hospitals were visited and caselogs and patient charts were manually reviewed to extract data. Multivariate logistic regression was used to determine predictors of local anesthesia use. We designed a survey instrument to assess the perceptions of physicians and anesthetists regarding different types of anesthesia for inguinal hernia repair. The survey was designed by a Ghanaian surgeon, reviewed by all co-authors, and tested prior to implementation using a sample (n = 8) of clinicians having similar practices to those of the survey population. Of 70 clinicians, 66 responded, yielding a response rate of 94%.

**Results:**

8080 patients underwent hernia repair of which 37% were performed under local anesthesia, while the majority, 60%, were performed under regional anesthesia. Negative predictors of local anesthesia were emergent repair (OR = 0.258, p < 0.001), surgery performed at a teaching hospital (OR = 0.105, p < 0.001), and bilateral hernia repair (OR = 0.374, p < 0.001). 1,839 (22.8%) of IH repairs were done on patients age 65 or older and RA was most frequently used among the elderly population (57.8%), while local anesthesia was used 39.5% of the time. Sixty-six clinicians participated in the survey with the majority reporting that local anesthesia requires fewer staff, less equipment, has a shorter recovery, is more cost-effective, and might be safer for patients. However 66% were unfamiliar with or incorrectly perceived international guidelines.

**Conclusion:**

To our knowledge, this study is the largest assessment of anesthesia use for inguinal hernia repair in an LMIC. Although the selection of anesthetic technique should be guided by a patient’s general health, the anatomy of the hernia, and clinician judgment, local anesthesia appears to be underutilized in northern Ghana. Survey responses demonstrate high rates of unfamiliarity or incorrectly perceived evidence-based guidelines. Future research should assess how education on the benefits and technique of local anesthesia administration may further increase rates for inguinal hernia repair, especially for older patients.

## Introduction

Inguinal hernia (IH) repair is one of the most commonly performed surgeries worldwide and the second most common major surgery in district hospitals in Ghana [1]. IH is one of the highest priority surgical conditions due to the high public health burden of IHs and the low cost of repair [2]. The surgery must be performed in a safe, cost-effective manner to justify the widespread use. This is true for all aspects of the operation, including both surgery and anesthesia. For planned IH repair, options for anesthesia include local infiltration of anesthesia (LA), regional anesthesia (RA), and general anesthesia (GA). An ideal anesthetic technique is first and foremost safe, but also is easy and efficient for surgeons and anesthetists, provides good intraoperative analgesia for patients, facilitates early discharge, is cost-effective, and is associated with few complications. The choice of anesthesia for elective open hernia repair may depend on several factors, including local capability, patient or surgeon preferences, and feasibility given patient comorbidities and the characteristics of the hernia.

The best anesthesia for open IH repair has been debated since the Shouldice Hospital [3] and then Lichtenstein Hernia Institute [4] demonstrated the feasibility of repair under LA. Since that time, LA has been shown to have many benefits: less patient-reported pain in the early post-operative period [5,6], no difference or greater patient satisfaction in the first postoperative week [7–10], and no difference in number of patients who would choose a different kind of anesthesia in the future [9,11]. LA is also the most cost-effective option [7,12], can be safely performed with little monitoring [13], and leads to faster postoperative recovery and discharge [6,7,11,14]. Most hernia centers use almost exclusively LA due to its advantages [13,15,16,17]. Reflecting evidence of the benefits of LA, international guidelines and statements from hernia societies recommend LA for most repairs along with either caution or a recommendation against the use of RA [18–21].

LA might be safer for patients. RA, which generally includes spinal, epidural, or paravertebral approaches, carries an increased risk of urologic complications [5,22–24] along with higher rates of myocardial infarction, pneumonia, and venous thromboembolism in elderly patients [24]. LA leads to less disturbance in pulmonary function compared to RA and GA [25] and is associated with a lower overall mortality risk for both elective and emergency surgery [26]. The safety profile of LA may be especially important in low- and middle-income countries (LMICs) that do not have access to resources that have made the delivery of anesthesia safer in high-income countries (HICs) [27]. While not specific to IH repair, rates of avoidable anesthesia-associated mortality in LMICs are 10–1,000 times greater than in HICs [27]: 1:3000 cases in Zimbabwe [28], 1:1900 in Zambia [29], 1:500 in Malawi [30] and 1:150 in Togo [31].

Ghana is a lower middle-income country, and the northern part of Ghana is one of the poorest areas with the worst access to surgical services [32]. Currently all anesthesia is provided by non-physician anesthetists. Previous groups demonstrated a 22% rate of LA use for IH repair in northern Ghana [33]. At that time Ghanaian physicians used LA less than visiting surgeons (16 vs 28%, respectively). More recently Operation Hernia demonstrated the viability and cost-effectiveness of using LA, using this technique for 72% of open hernia repairs with the remaining patients being either children or having hernias too large for LA [34]. Yenli et al. demonstrated the feasibility of using LA in rural Ghana, using LA for 71.2% of IH repairs over a three year period by the ApriDec Medical Outreach Group [35].

Although rates of LA have been documented for outreach groups, little is known about its use in typical surgical practice across northern Ghana. The purpose of this study is to assess the type of anesthesia used for hernia repair in the northern part and transitional zone of Ghana and identify positive and negative predictors for the use of LA. In addition, we sought to assess the perceptions of the different types of anesthesia among physicians and anesthetists in northern Ghana to better understand knowledge and perceived barriers to the use of LA.

## Materials and methods

### Review of operative case logs

In order to identify our study population operative logbooks from January 2013 through December 2017 were reviewed for all inguinal hernia (IH) repairs in the above-mentioned regions. This method of obtaining operative volume has been validated in sub-Saharan Africa, with 99% accuracy of capturing operative volume [36]. Throughout 2017 co-investigators and trained research assistants performed a retrospective review during in-person visits to participating hospitals. Logbooks are standardized by the Ghana Health Services and include: age, gender, diagnosis including hernia type, procedure performed, date of surgical procedure, surgeon provider, anesthesia provider, anesthesia type, and elective versus emergent procedure. Anethesia type was most often recorded as LA, RA, or GA, but when further characterized, spinal, epidural, or paravertebral approaches were considered RA while a transversus abdominus plane block with local infiltration was categorized as LA. When all variables were not available, a chart review was conducted to complete data extraction. Cases from more than 95% of hospitals in the northern part (Upper East, Upper West and Northern regions) of Ghana and 89% of hospitals in the transitional zone (Brong-Ahafo regions) were captured. All male patients above 17 years undergoing open IH repair were included. The benefits of local anesthesia (LA) for children has not been demonstrated, and they were excluded from analysis. Female patients represented < 1% of patients and were also excluded.

### Categorization of hospitals

Forty-one public hospitals in the Upper East, Upper West, Northern and Brong-Ahafo regions of Ghana were included in the study. Hospitals were categorized into teaching, regional and district based on World Health Organization criteria [37]. A “teaching” hospital is defined as a hospital that provides medical education and included only Tamale Teaching Hospital, in the Northern Region. A “regional” hospital or Level 3 Referral hospital is defined as a hospital of 300 or more beds with basic intensive care facilities. In our sample, this included Sunyani Regional Hospital in Brong Ahafo, Tamale Regional Hospital in the Northern Region, Bolgatanga Regional Hospital in the Upper East Region and the Wa Regional Hospital in the Upper West Region. “District” or Level 2 hospitals have up to 300 beds with adequately equipped major and minor operating theatres. The majority of hospitals in our study fit into this category.

### Categorization of surgeons

Not all physicians performing surgical procedures in Ghana receive formal surgical training. The Ghana College of Physicians and Surgeons provides surgical training, which includes a membership after three years. Physicians that underwent this in-country training or surgical residency abroad were considered surgeons, while all other physicians performing surgical procedures were considered non-surgeon physicians.

### Categorization of hernias

Types of IH were further categorized using the Kingsnorth Criteria: H1 groin only and reduced spontaneously, H2 groin only and reduced with manual pressure, H3 reducible inguinoscrotal, H4 irreducible inguinoscrotal [38]. Further characterization included recurrent, bilateral, strangulated and obstructed hernias. Recurrent was defined as a second hernia after previous surgical repair. Irreducible hernias were further categorized into strangulated hernias, indicating absent blood flow to the hernia contents and obstructed hernias in which the lumen of herniated bowel is obstructed.

### Statistical analysis

Descriptive statistics were used to study patient characteristics and determine rates of IH repair by region and district. Multivariate logistic regression was performed to determine predictors of the use of LA. Predictors of LA in our model included the type of hospital where the IH repair was conducted, whether a surgeon performed the operation, whether IH repair was performed using mesh, and characteristics of the hernia. Hospital type included teaching, regional, and district. IH repair being performed in a district hospital was used as the comparator to teaching and regional hospitals.

Odds ratios are presented to measure the strength of association of our predictor variables for the use of LA. Statistical significance was set at p < 0.05 and 95 percent confidence intervals are presented in our regression table. All statistical analysis was done using Stata 15.1 (StataCorp. College Station, TX). IH repair maps were developed using the SPMAP module on Stata and further edited using Microsoft Powerpoint (Microsoft Corporation, Santa Rosa, CA).

### Survey of physicians and anesthetists

We designed a survey instrument to assess the perceptions of physicians and anesthetists regarding different types of anesthesia for IH repair. The survey design specifically asked about perceived benefits of anesthesia where evidence supports a certain mode of anesthesia delivery. The survey instrument was initially designed by a senior, practicing Ghanaian surgeon who was from the region and understood the clinics and culture. The survey was then reviewed by all co-authors, including additional senior and physicians knowledgeable in inguinal hernia repair in the region. Originally 30 questions, the group refined the survey to 18 questions. Our survey was further revised based on cognitive interviews among the co-authors group. We then modified the survey and prepared a user-friendly interface using Google Forms, a web-based survey-hosting program (Alphabet Inc., Mountain View, CA). The survey was tested prior to implementation using a sample (n = 8) of Ghanaian surgeons, non-surgeon physicians and anesthetists in the region, having similar clinical practices to those of the survey population.

The questionnaire asked whether LA, RA or GA was most convenient for surgeons and anesthetists and which provided the best pain control for patients. Clinicians were asked which form of anesthesia requires the fewest staff, least monitoring, shortest time to recovery, safest and most cost-effective methods, along with an estimated cost of anesthetic medications. Clinicians were asked which type of anesthesia is recommended by international guidelines (LA, RA, GA, no recommendations are made, or not sure). Finally, clinicians were asked which type of anesthesia should be recommended for primary hernia repair in Ghana. The survey is provided in S1 File.

The survey was sent by mobile phone and e-mail to 70 clinicians known to conduct or participate in IH repair. The survey remained open from February 14, 2018 to March 14, 2018. Two weeks after the first request, reminder messages were sent at one-week intervals to all participants. The response rate was 66 clinicians, yielding a response rate of 94%. No incentives were provided for participation.

### Ethical review

This study was exempted from IRB review by the Tamale Teaching Hospital, as it involves less than minimal risk to patients and clinicians involved. Minimal risk was defined as “the probability and magnitude of harm or discomfort anticipated in the research are not greater in and of themselves than those ordinarily encountered in daily life or during the performance of routine physical or psychological examinations or tests.” No patient-identifying information was collected and all data was stored on encrypted computers by co-researchers. The survey respondents did not provide any identifying information and consent was obtained by voluntary participation.

## Results

**Fig 1:**
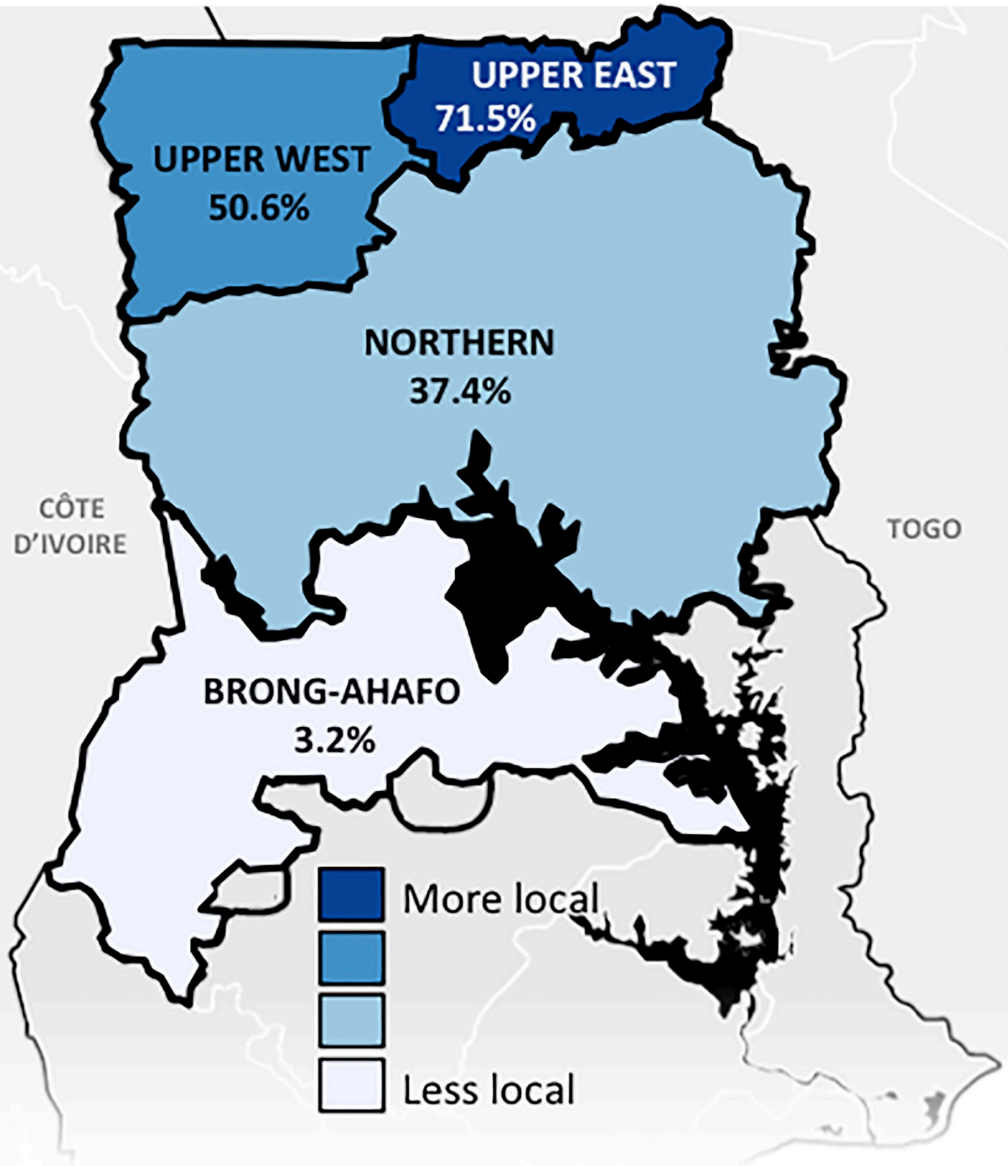
Rates of local anesthesia use for inguinal hernia repair by region.

8080 male patients in the study hospitals underwent open IH repair in the northern part and transitional zone of Ghana during the years 2013–2017. The average age at repair was 48.5 (Table 1). The majority of cases were performed at district hospitals (84%). Simple inguinal hernias represented 66% of repairs, inguinoscrotal 28%, while bilateral hernias accounted for 6%. Most IH were repaired non-urgently (93%) and recurrences from remote repair represented 0.77% of cases. Suture repair was the most common repair (85%) while mesh repair was performed in 15% of patients. The operation was most often performed by non-surgeon physicians (66%) while the remaining were performed by surgeons (34%). RA was the most common form of anesthetic administered (60%), followed by LA (37%) and then GA (3%). Rates of LA use for open IH repair were highest in the Upper East Region (71.5%), followed by Upper West Region (50.6%), Northern Region (37.4%), and Brong-Ahafo Region (3.2%) (Fig 1). Hospital volume and % LA were not correlated with a correlation coefficient 0.057 (p = 0.72).

**Table 1:** Demographics of patients undergoing inguinal hernia repair in northern Ghana (n = 8080).

Mean Age	48.5 years
**Hospital type**	
District: n = 36 hospitals, mean 189 repairs (range 15–304)	84%
Regional: n = 4 hospitals, mean 283 repairs (range 150–372)	14%
Teaching: n = 1 hospital, 162 repairs	2%
**Hernia type**	
Right	41%
Right inguinoscrotal	19%
Left	25%
Left inguinoscrotal	9%
Bilateral	6%
**Repair type**	
Suture repair	85%
Mesh repair	15%
**Physician performing surgery**	
Non-surgeon physicians	66%
Surgeon	34%
**Anesthesia type**	
Regional anesthesia	60%
Local anesthesia	37%
General anesthesia	3%

1,839 (22.8%) of IH repairs were done on patients age 65 or older. RA was most frequently used among the elderly population (57.8%), while LA was used 39.5% of the time. The type of anesthesia used for the population 65 and older was nearly identical to the type of anesthesia less than 65 years.

Multivariate logistic regression analysis was conducted to estimate the probability of IH repair under LA from a set of binary predictor variables and controlling for patient age (Table 2). All but one predictor variables were negatively associated with LA. The strongest negative predictors of LA were patients undergoing IH repair emergently (OR = 0.258, 95% CI = 0.198, 0.337), operations performed at a teaching hospital (OR = 0.105, 95% CI = 0.051, 0.216) compared to a district hospital, and bilateral IH repair (OR = 0.374, 95% CI = 0.387, 0.522). Other statistically significant negatively associated predictor variables were inguinoscrotal hernia (OR = 0.629, 95% CI = 0.564, 0.701), and operations performed at a regional hospital (OR = 0.449, 95% CI = 0.387, 0.522) compared to a district hospital. The only positive predictor of LA in our model was IH repair being performed by a surgeon (OR = 1.253, 95% CI = 1.133, 1.386). IH being recurrent or irreducible were not statistically significant predictors of LA. The likelihood ratio chi-square test statistic indicated that the overall model was significant (LR ^2^_(10)_ = 537.761 (p<0.001)). Our model correctly predicted 62.8% of the outcome variable (Count R^2^ = 0.628) with a positive predictive value of 50.5% and a negative predictive value of 64.3%.

**Table 2:** Multivariate logistic regression model of local anesthesia.

Predictor Variable	OR	p-value	95% CI
Age	1.001	0.280	(0.999, 1.004)
Emergency	0.258	0.000	(0.198, 0.337)
Surgeon	1.253	<0.001	(1.133, 1.386)
Recurrent	0.640	0.170	(0.338, 1.211)
Inguinoscrotal	0.629	<0.001	(0.564, 0.701)
Bilateral	0.374	<0.001	(0.297, 0.470)
Teaching hospital^a^	0.105	<0.001	(0.051, 0.216)
Regional hospital^a^	0.449	<0.001	(0.387, 0.522)
Irreducible^a^	0.904	0.691	(0.549, 1.488)
N	8080		
Count R^2^	0.628		
LR c^2^	537.761	<0.001	

^**a**^The comparator hospital location for Teaching and Regional is District hospital. Irreducible IH includes strangulated, obstructed, and incarcerated. Coefficients are presented as odds ratios with 95% confidence intervals reported in parenthesis.

**Fig 2:**
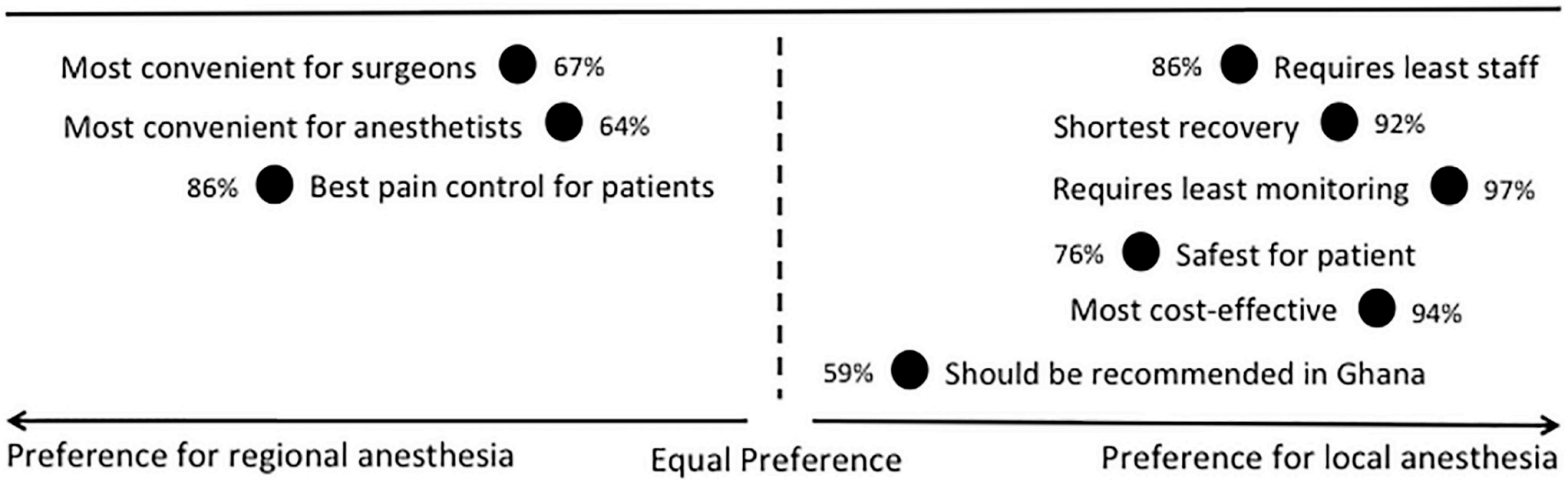
Perceptions of local and regional anesthesia among physicians and anesthetists in northern Ghana. Based on a survey of 66 clinicians in northern Ghana.

Of the 70 clinicians invited to participate in the survey, 66 responded, yielding a response rate of 94%. Perceptions of different types of anesthesia for the use of primary IH repair among physicians and anesthetists in northern Ghana are summarized in Fig 2. Of 70 contacted, 66 clinicians responded, yielding a response rate of 94%. Thirty-five (53%) respondents were non-surgeon physicians, 22 (33%) were anesthetists, and 9 (14%) were surgeons. 45% were from the Northern Region, 26% from the Brong-Ahafo Region, 15% from the Upper West Region, and 14% from the Upper East Region. The majority (65%) primarily practice in district hospitals, followed by regional hospitals (21%) and teaching hospitals (14%). 65% reported using RA most often with the remaining 35% using LA most often. The majority of the clinicians (67%) reported that RA is more convenient for surgeons, while 65% thought RA is more convenient for the anesthesia provider. 86% reported that RA results in the best pain control for patients, while 12% and 2% felt that LA and GA provided better pain control, respectively. LA was perceived to be associated with requiring fewer staff (86%), a shorter recovery (92%), and requiring less monitoring (97%). The majority of clinicians (76%) felt that LA is the safest type of anesthesia for IH repair and 94% thought it is the most cost-effective. The median estimated cost of LA was $6 USD (IQR 4–13) while RA was $25 USD (IQR 16–38). When asked what type of anesthesia international guidelines recommend, 38% chose LA, 33% were not sure, 24% thought RA, and 5% thought no recommendations were made. When asked what type of anesthesia should be recommended for IH repair in Ghana, 59% suggested LA while 41% recommended RA.

Clinicians in the Upper East (60%) and Upper West (44.5%) Regions had the highest awareness that LA is recommended by international guidelines (Table 3). 54.3% of non-surgeon physicians and 44.5% of surgeons thought LA was recommended by international guidelines while 45.5% of anesthetists thought RA was recommended.

**Table 3:** Understanding of international guidelines for primary inguinal hernia repair by region and provider.

	% reporting LA ^a^	% reporting RA	% unaware
**Overall** (n = 66)	38%	24%	38%
**By region**
Upper West (n = 10)	60%	0%	40%
Upper East (n = 9)	44.5%	0%	55.5%
Northern (n = 31)	25.8%	35.5%	38.7%
Brong-Ahafo (n = 17)	41.2%	29.4%	29.4%
**By provider**
Non-surgeon physician (n = 35)	54.3%	14.3%	31.4%
Surgeon (n = 9)	44.5%	11%	44.5%
Anesthetist (n = 22)	9%	45.5%	45.5%

^a^ Recommended by HerniaSurge Group, British Hernia Society, European Hernia Society, and Danish Hernia Database Group [18–21].

## Discussion

The selection of anesthetic technique should be guided by a patient’s general health, the anatomy of the hernia, and clinician judgment. However, several studies have demonstrated the superiority of LA, which should be the first considered method for primary, uncomplicated open IH repair. Both the Operation Hernia and ApriDec surgical outreach groups have demonstrated the feasibility of high rates (72 and 71.2%) of LA in Ghana [34,35]. The majority of patients (55%) undergoing IH repair in northern Ghana receive RA while LA was used only 42% of the time. The rate of LA represents an almost two-fold increase from 2006 [33], but is roughly half the rate achieved by outreach groups. Based on standards set by international guidelines, LA appears to be underutilized in northern Ghana.

Knowledge of international guidelines likely contributes to the low adoption of LA. In areas where more clinicians are aware of international recommendations (Upper East and Upper West) for LA, rates of LA for IH repair are highest. Interestingly, these are the poorest areas in Ghana where resources are most scarce, and the high rates may be secondary to outreach programs or the education provided by these clinicians. In addition, non-surgeon physicians and surgeons are most familiar with the guidelines and, indeed, IH repair being performed by a surgeon was a positive predictor of LA. The majority of clinicians (62%) are unaware of, or incorrectly perceived international guidelines. The British Hernia Society, HerniaSurge Group, Danish Hernia Society, and European Hernia Society all recommend avoiding RA [18–21]; however 45.5% of anesthesthetists thought RA was recommended, while 45.5% reported being unaware of international guidelines. Multiple groups specifically recommend against using RA in patients above the age of 65 [21,24]. Although GA is mentioned as an alternative to RA, many hospitals in northern Ghana have limited access to an anesthestic machine, oxygen, and monitoring equipment required for GA, and this method should not be recommended for elective cases in this setting. Elderly patients in northern Ghana had similar rates of RA compared to younger patients and, although we did not ask clinicians, we hypothesize that these recommendations are not appreciated. We posit that clinicians should be aware of guidelines—the decision to follow them should then be individualized to the clinicians’ interpretation of the strength of the evidence, their own experience, and the clinical scenario. Improved circulation of international guidelines and increased training on the use of LA, especially in the Northern and Brong-Ahafo Regions, may increase rates of LA use for IH repair.

Generally, the perception of the benefits of LA by Ghanaian physicians and anesthetists regarding reflect those found in the available literature: LA requires fewer staff, less equipment, has a shorter recovery, is more cost-effective, and might be safer for patients. However, the majority (86%) of anesthetists and surgeons think that pain control is superior under RA. This perception might reflect a lack of training as suggested in one survey: “Regional or general anesthesia is used by most anesthetists due to a lack of adequate knowledge and skill in local anesthesia block techniques.” Surgery being performed at a teaching hospital was a negative predictor for the use of LA, which may reflect the lack of experience of trainees. The need for training is also reflected in guidelines such as the HerniaSurge Group’s statement: “Local anesthesia in open repair has many advantages, its use is recommended provided the surgeon is experienced in this technique [21].” Although physicians perceive that RA or GA might make the operation easier to perform, multiple publications have shown that training can make the use of LA practical for physicians, anesthesia providers, and patients [3,13,15,39,40]. Further, the learning curve in the use of LA for IH repair might be short, and a variety of methods are published [15,41–46].

Not all patients are good candidates for LA including those with giant inguino-scrotal hernias otherwise known as “wheelbarrow” or “below the knees” hernias. Operation Hernia also reported patients having hernias too large for local anesthesia [34]. Similarly Yenli et al. reported reduced rates of LA for inguinoscrotal hernias [35]. Bourgouin et al. reported good results for LA in small and medium-sized hernias in sub-Saharan Africa, but recommended ultrasound-guided blocks for best results in larger hernias [47]. Ultrasound-guided blocks are not being used in northern Ghana, but should be considered. Reflecting the experience of other groups, repair of an inguinoscrotal hernia was a negative predictor for the use of LA in northern Ghana. One survey response highlighted this: “Most hernias in Ghana are large and have undergone recurrent obstructions with adhesions around the cord and content of the sac. Using local anaesthesia may cause pain.” Bilateral IH repair, a negative predictor of LA use, might also represent a challenge for adequate pain control with LA, as more area needs to be anesthetized with a limited amount of anesthetic agent. Even considering that some inguinoscrotal and bilateral hernias may necessitate RA or GA, our rate of combined inguinoscrotal and bilateral hernias (34%) was well below the rate of RA (60%), suggesting an area for improvement. While the benefits and feasibility of LA for small and medium-sized hernias is clear, more study for larger, inguinoscrotal and bilateral hernias in LMICs is needed. Clinicians should strive to repair primary, uncomplicated IH using LA in 70% of cases–this rate balances the proven benefits of LA and specific patient factors that might necessitate another method of anesthesia. Further, this practice is well supported by international guidelines and the feasability of this rate has been proven by two prior studies in this region [35,36].

Not only may this study guide the practice of physicians and anesthetists in Ghana and other LMICs, it may reinforce the existing scientific data and recommendations for clinicians in HICs. Rates of LA for elective, open IH repair in well-resourced areas are not in accordance with guidelines and range from 18 to 66% [48–49]. It is likely that a survey about perceptions of guidelines in HICs would yield similar results as Ghana: physicians and anesthesia providers are unfamiliar and would benefit from education.

There are multiple limitations to this study. The accuracy of our findings are limited to the handwritten operative logbooks, which may have incorrect or missing data. We have excluded private and hospitals and clinics because data collection in these facilities vary from the logbooks mandated by the Ghanaian government. However, our data covers the majority of public hospitals in the regions studied. Emergency surgery was a negative predictor for the use of LA: due to the limitations of our data collection, we are unable to assess if concurrent laparotomy and bowel resections were needed for emergent cases, which might require GA. Less than 1% of patients were women, a finding not consistent with our own experience. We are unsure how our method of data collection could underestimate the number of women receiving inguinal hernia repair. Rates of LA were similar (40%). The rate and practices of hernia repair for women in Ghana warrants further study. Finally, our survey regarding perceptions of anesthesia included limited sample of physicians and anesthetists. We are uncertain how this sampling would bias the results. Our high response rate of 94%, however, is an indirect measure of an effective survey design.

## Conclusions

To our knowledge, this study is the largest assessment of anesthesia use for IH repair in an LMIC and may help target interventions in northern Ghana and serve as a guide for other LMICs. Although the rates of LA use for IH repair is increasing in northern Ghana, there still is room for improvement. Further, knowledge of evidence-based guidelines among clinicians in northern Ghana may lead to increased rates of LA for primary hernia repair.

## Supporting information

S1 File (PDF)
