# Supplementary material for: Local anesthesia underutilized for inguinal hernia repair in northern Ghana
Source: PLoS One. 2018 Nov 21;13(11):e0206465. doi: 10.1371/journal.pone.0206465 (PMC6248905; doi:10.1371/journal.pone.0206465)
Supplement: S1 File — (PDF) [file pone.0206465.s001.pdf]

Anesthesia in northern Ghana

In the repair of inguinal (groin) hernia, the selection of anesthesia type should be guided by a patient's general health, the anatomy of the hernia, and clinician judgment. The purpose of this questionnaire is to assess which type of anesthesia should be standardly used in uncomplicated, Kingsnorth H1 (groin only, reduces spontaneously on lying down), H2 (groin only, reduces completely with gentle manual manipulation), and H3 (inguinoscrotal, reducible with manual manipulation) hernias.

Please answer the following questions honestly. We will not collect any identifying information other than your position and location of practice.

There are 18 questions in this survey. By returning this questionnaire, you are giving your consent and authorization to participate in this study.

Q1: What is your position?
1. Medical officer
2. Surgeon
3. Anesthetist
4. Urologist
5. Other:

Q2: In which region do you primarily practice?
1. Brong-Ahafo Region
2. Northern Region
3. Upper East Region
4. Upper West Region
5. Other:

Q3: At what type of hospital do you primarily practice?
1. District hospital
2. Regional hospital
3. Teaching hospital

Q4: During primary hernia repair, which type of anesthesia do you most often use?
1. Local anesthesia
2. Regional (or spinal) anesthesia
3. General anesthesia

Q5: What type of anesthesia is most convenient for surgeons during primary hernia repair?
1. Local anesthesia
2. Regional (or spinal) anesthesia
3. General anesthesia

Q6: What type of anesthesia is most convenient for anesthetists during primary hernia repair?
1. Local anesthesia
2. Regional (or spinal) anesthesia
3. General anesthesia

Q7: What type of anesthesia is best for patient's pain control during primary hernia repair?
1. Local anesthesia

2. Regional (or spinal) anesthesia
3. General anesthesia

Q8: What type of anesthesia requires the fewest staff during primary hernia repair?
1. Local anesthesia
2. Regional (or spinal) anesthesia
3. General anesthesia

Q9: What type of anesthesia has the shortest time of recovery after primary hernia repair?
1. Local anesthesia
2. Regional (or spinal) anesthesia
3. General anesthesia

Q10: What type of anesthesia requires the least monitoring equipment during and after primary hernia repair?
1. Local anesthesia
2. Regional (or spinal) anesthesia
3. General anesthesia

Q11: What type of anesthesia is safest for patients during primary hernia repair?
1. Local anesthesia
2. Regional (or spinal) anesthesia
3. General anesthesia

Q12: What type of anesthesia is most cost-effective for primary hernia repair?
1. Local anesthesia
2. Regional (or spinal) anesthesia
3. General anesthesia

Q13: Please estimate the cost of medications (in Ghanaian cidi) for local anesthesia for primary hernia repair.

Q14: Please estimate the cost of medications (in Ghanaian cidi) for regional (spinal) anesthesia for primary hernia repair.

Q15: Please estimate the cost of medications (in Ghanaian cidi) for general anesthesia for primary hernia repair.

Q16: What type of anesthesia do international guidelines recommend for primary hernia repair?
1. Local anesthesia
2. Regional (or spinal) anesthesia
3. General anesthesia
4. No recommendations are made
5. Not sure

Q17: In Ghana, what type of anesthesia should be recommended for primary hernia repair?
1. Local anesthesia
2. Regional (or spinal) anesthesia
3. General anesthesia
4. Not sure/no preference

Q18: Do you have any other comments about anesthesia for primary hernia repair?
